# Prevalence and associations of psychoactive substance use among male supportive staff members in a tertiary care hospital of Sri Lanka

**DOI:** 10.1192/bjo.2021.778

**Published:** 2021-06-18

**Authors:** Chathurie Suraweera, Iresha Perera, Priyanka Rupasinghe, Janith Galhenage

**Affiliations:** 1 Professorial Psychiatry Unit, National Hospital of Sri Lanka; 2 Department of Paediatric Neurology, Lady Ridgeway Hospital for Children

## Abstract

**Aims:**

The study describes the prevalence and associated socio-demographic variables of psychoactive substance use among male supportive staff members at a tertiary care hospital in Sri Lanka.

**Method:**

A cross-sectional descriptive study was carried out among male supportive staff members of a tertiary care hospital in Colombo District, Sri Lanka by using a self-administered anonymous questionnaire. Participants were recruited using stratified cluster sampling in thirteen overseer divisions of the hospital. Anonymous questionnaires were collected into a sealed box and analysed using Statistical Package for Social Sciences 20.

**Result:**

The mean age of the 404 male staff members who participated in the study was 38.78(SD = 10.90) years and 71.5% were married. Among them 202 (49.1%) were educated up to grade 6-11 and 30 of them has had encounters with law in the past. Thirty of participants had history of psychoactive substance use in the family. Alcohol was used more than once a month by 127(30.9%) and more than once a week by 19(4.6%) individuals. Among other substances, tobacco, beetle and beedi were used by 104(25.3%), 78(19.0%) and 18(4.4%) respectively at least once a month. Further, 22(5.3%), 20(4.8%), 7(1.7%) and 7(1.7%) participants used Mava, Cannabis, Methamphetamine and Thool respectively at least less than once a month. Heroin, Tramadol and Morphine were used by two individuals at least less than once a month. Among substance using participants, 132 wished to cut down their habit. Most commonly identified (14.1%) adverse consequence was financial issues secondary to psychoactive substance use. Eleven (4.5%) staff members used the substance at hospital. Alcohol use was associated with age more than 35 years (p = 0.039) and history of forensic involvement (p = 0.038). Tobacco(p = 0.000), beetle (p = 0.056), Cannabis (p = 0.000) and mava (p = 0.015) use were significantly associated with positive forensic history. Supportive staff members’ alcohol and cannabis use was associated with tobacco (p = 0.000, p = 0.000) and beetle use (p = 0.001, p = 0.049). Mava use was associated with alcohol (p = 0.060) use in addition to tobacco (p = 0.020) and beetle use (p = 0.008).

Binomial logistic regression revealed alcohol use and beetle use were associated with the number of children in family and above associations.

**Conclusion:**

Commonest psychoactive substance consumed by supportive staff members were alcohol, tobacco, beetle, Cannabis and Mava in descending order of frequency. Forensic history was significantly associated with substance use. True prevalence of substance use can be higher than these values.

